# Zinc Sulfate Alleviates Olanzapine Induced Alteration in Hepatic Protein Patterns and Antioxidant Defense System in Rats

**DOI:** 10.1007/s12011-025-04673-3

**Published:** 2025-05-30

**Authors:** Wael Mahmoud Aboulthana, Sherif Abdelmottaleb Moussa, Samir Wassef Aziz, Amal Gouda Hussien, Enayat A. Omara, Samir A. E. Bashandy

**Affiliations:** 1 Biochemistry Department, Biotechnology Research Institute, National Research Centre, 33 El Bohouth St, P.O. 12622, Dokki, Cairo, Egypt; 2 Pathology Department, National Research Centre, 33 El Bohouth St, P.O. 12622, Dokki, Cairo, Egypt; 3 Pharmacology Department, National Research Centre, 33 El Bohouth St, P.O. 12622, Dokki, Cairo, Egypt

**Keywords:** Olanzapine, Antioxidants, Electrophoresis, Isoenzymes, Dynamic motion of hemoglobin

## Abstract

One of the atypical antipsychotic drugs is olanzapine (OLZ). Enhancing metabolic and detoxifying activities requires zinc (Zn). Thus, the objective of the current study was to determine if Zn supplementation might effectively reduce hepatic lesions induced by OLZ. The oxidative stress, inflammatory, and fibrotic indicators of the liver tissues were evaluated, and electrophoretic methods were used to analyze the native protein and isoenzyme patterns. Furthermore, the liver tissues were examined histopathologically. The dynamic motion of the extracted hemoglobin was also examined. In addition to the serious damage identified histopathologically, our results show that OLZ treatment altered the liver tissue’s antioxidant and inflammatory indicators. The zinc sulfate (ZnSO_4_) salt solution, given in a dose-dependent fashion, reduced these changes. Additionally, the ZnSO_4_ salt solution enhanced the natural protein, lipid, and calcium moieties of the protein pattern that were electrophoretically detected and changed by OLZ. In terms of the isoenzyme patterns, the ZnSO_4_ salt solution reduced the electrophoretic catalase (CAT), peroxidase (POX), and β-esterase (β-EST) isoenzyme patterns that were hampered by OLZ in a dose-dependent manner. Rats treated with OLZ in addition to ZnSO_4_ showed an increase in heme-heme interactions, suggesting that zinc therapy stabilized oxyhemoglobin. This promotes a more effective folding process that improves the use of oxygen. A dosage of 100 mg/kg of ZnSO_4_ was shown to normalize physiological, histological, and biochemical markers. It also improved interactions between heme molecules.

**Supplementary Information:**

The online version contains supplementary material available at 10.1007/s12011-025-04673-3.

## Introduction

Olanzapine (OLZ) and other atypical antipsychotic drugs are frequently prescribed to treat psychotic disorder. They are considered the prototype of the second generation. OLZ has also been used as monotherapy or in combination with antidepressants to treat depressive disorders with or without psychotic symptoms [1].

The cytochrome P450 system primarily converts OLZ into inactive metabolites in the liver, which is the primary location of OLZ metabolism [2]. Both high and low dosages of OLZ are sufficient to damage rat livers at the cellular level in histological and stereological studies. Therefore, while it is effective for stress-related mental illnesses, further studies are needed to understand its effects on the liver [3]. Given the increased risk of death and consequences from obesity, it is important to emphasize that OLZ is associated with several metabolic issues, including weight gain, hyperglycemia, and hypertriglyceridemia, all of which should be treated with extreme caution [4].

The majority of studies on the metabolic abnormalities induced by OLZ have been conducted in normal experimental animals due to the limitations of the schizophrenia model. Further investigations are needed to determine if the schizophrenia phenotype affects the metabolic anomalies induced by OLZ [5]. Inflammation and oxidative stress occur with the administration of OLZ. High blood pressure, impaired glucose tolerance, and hyperlipidemia are examples of metabolic comorbidities that significantly impact and contribute to metabolic dysfunction. They play a crucial role in the development of metabolic diseases [6, 7].

Zinc (Zn) is one of the most important trace elements in all tissues, secretions, and liquids. It is essential for metabolism, detoxification, growth, development, signaling pathways, and gene regulation [8]. It also carries out a number of biological functions, including protein synthesis, proliferation, redox equilibrium, and antioxidant activities, by maintaining the integrity of the cell membrane, increasing the amount of metallothionein (MT), or decreasing calcium influx [9].

Zn is particularly important for cellular function as it is an essential part of many proteins, such as structural proteins, transcription factors, and metalloenzymes. Apart from its role as a component of several enzymes involved in cell division and macronutrient metabolism, Zn is also considered a cofactor [10]. Low Zn levels can activate rat hepatocytes, which are vulnerable to oxidative stress. It has been discovered that Zn provides protection against toxicant-induced liver damage [11].

Proteins that scavenge superoxide and hydroxyl radicals can be activated by Zn [12]. This is why the current study was conducted to demonstrate how Zn supplementation protects rats against oxidative hepatic lesions, changes in hepatic protein patterns, and alterations in hemoglobin mobility induced by OLZ.

## Materials and Methods

### Experimental Design

In the Animal House of the National Research Center in Giza, Egypt, thirty-two (32) adult male Wistar rats weighing between 120 and 150 g were kept in a well-regulated environment. The rats were fed ad libitum with a standard chow diet purchased from local feed production companies following international specifications for food ingredients for rodents as presented in Supplementary Table 1. They were given free access to tap water. They were divided randomly into four groups (eight rats per group).

*The control group (G1)* received 1 mL of distilled water daily (OLZ vehicle), along with normal food and distilled water for 8 weeks. *The OLZ-treated group (G2)* was orally administered OLZ at a dose of 10 mg/kg body weight for 8 weeks, following the study conducted by Ardıç et al. [13]. *The OLZ-* + *ZnSO*_*4*_*-treated groups (G3 and G4)* were orally treated with OLZ as in G2 and ZnSO_4_ at doses of 50 and 100 mg/kg, respectively for 8 weeks as proposed by Ebaid et al. [14].

### Collection of Blood Samples and Tissues

Eight weeks after treatment, the rats were starved for around 12 h. Rats in all groups were anesthetized using xylazine and ketamine (20 and 50 mg/kg, intraperitoneally, respectively). The liver tissues of the animals were removed following cervical dislocation and cleaned in ice-cold saline. Using the Tissue Master TM125 (Omni International, USA), the tissue was homogenized in potassium phosphate buffer (pH 7.4) and centrifuged for ten minutes at 3000 rpm. For histological analysis, a portion of the autopsied tissue was stored in a 10% neutral buffered formalin solution. The clean supernatants from the biochemical assays were stored at − 80 °C for storage.

### Biochemical Assays in Liver Tissues Homogenates

#### Oxidative Stress Markers

In the clear supernatants of the liver tissue homogenates, oxidative stress indicators such as reduced glutathione (GSH) [15] and total antioxidant capacity (TAC) [16] were measured as µmol/g and mg/g tissue, respectively. Total protein carbonyl (TPC) [17] and lipid peroxidation products (LPO) [18] were measured and reported as nmol of reactive carbonyl compounds per mg protein of tissue and nmol/g wet tissue, respectively.

#### Fibrotic and Inflammatory Reactions Markers

The amount of the fibrotic marker hydroxyproline [19] was expressed as µg/mg of tissue. Additionally, the quantitative sandwich enzyme immunoassay (ELISA) technique was employed to test two commonly used inflammatory markers, interleukin-6 (IL-6) [20] and tumor necrosis factor-α (TNF-α) [21], and the results were represented as pg/g of tissue.

### Statistical Analysis

Results from five replicates are presented as mean ± standard error (SE). The data were statistically examined by a one-way analysis of variance (ANOVA) test using the Statistical Package for Social Sciences (SPSS for Windows, version 19.0), followed by the Bonferroni test as a post-hoc analysis. A “*p*” value less than 0.05 indicates a statistically significant difference between the groups being investigated.

### Histopathological Examination

The liver was preserved for 48 h in neutrally buffered formalin in order to be examined. After that, it was washed in distilled water, put through a grading sequence of alcohol processes, removed with xylene, and then imbedded in paraffin wax. The 5-μm-thick paraffin slices were stained with hematoxylin and eosin (H&E) [22]. A light microscope was used for inspecting for histopathological changes in the stained sections.

### Electrophoretic Assays

#### Native Protein Patterns

Following the homogenization of the liver tissues (with known weights) in extraction buffer, the mixture was centrifuged. To ensure that the protein concentrations in each well were the same, the Bradford method [23] was used to determine the protein concentration in the supernatants. The protein concentration was then diluted with sample loading dye according to the protein concentration. Vertical slab polyacrylamide gel electrophoresis was used to assay the protein bands, lipids, and calcium moieties of native proteins using Coomassie Brilliant Blue (CBB) [24], Sudan Black B (SBB) [25], and Alizarin Red “S” [26].

#### Isoenzyme Patterns

To identify the electrophoretic catalase (CAT) and peroxidase (POX) types, the native gel was treated with the particular conditioning buffer and hydrogen peroxide (H_2_O_2_) as a substrate. It was then stained with potassium iodide (KI) [27] and benzidine [28]. The native gel was colored with an iodine solution after being incubated in a soluble starch solution as a substrate to distinguish the electrophoretic α-amylase (α-Amy) types [29]. The β-esterase (β-EST) types were identified electrophoretically by coloring the gel with a reaction mixture that included Fast Blue RR and β-naphthyl acetate substrates, as suggested by Ahmad et al. [30].

#### Data Analysis

Quantity One software (Version 4.6.2) was used to analyze the colored bands to determine the relative mobilities (Rfs), band quantity (Qty), and band percentage (B%) of the electrophoretically separated bands. The percentages of genetic distances (GD%) and similarity indices (SI%) between each treated group and the control were computed using a method developed by Nei and Li [31].

### Extraction and absorption spectra of hemoglobin

Using a modified method described by Trivelli et al. [32], hemolysates of packed and cleaned erythrocytes were prepared. Under these conditions, the hemoglobin concentration was found to be between 2.9 and 3.4 × 10^−5^ M. The deoxyhemoglobin component was ignored since it is too small to be detected spectrophotometrically at this high dilution and under air-saturated conditions. A UV/VIS dual-beam spectrophotometer with kinetic measurements (model: DS5) from Edinburgh Instruments, UK, was used to generate HbO_2_ absorption spectra at 25 ± 1 °C.

## Results

### Oxidative Stress Markers

**Fig. 1:**
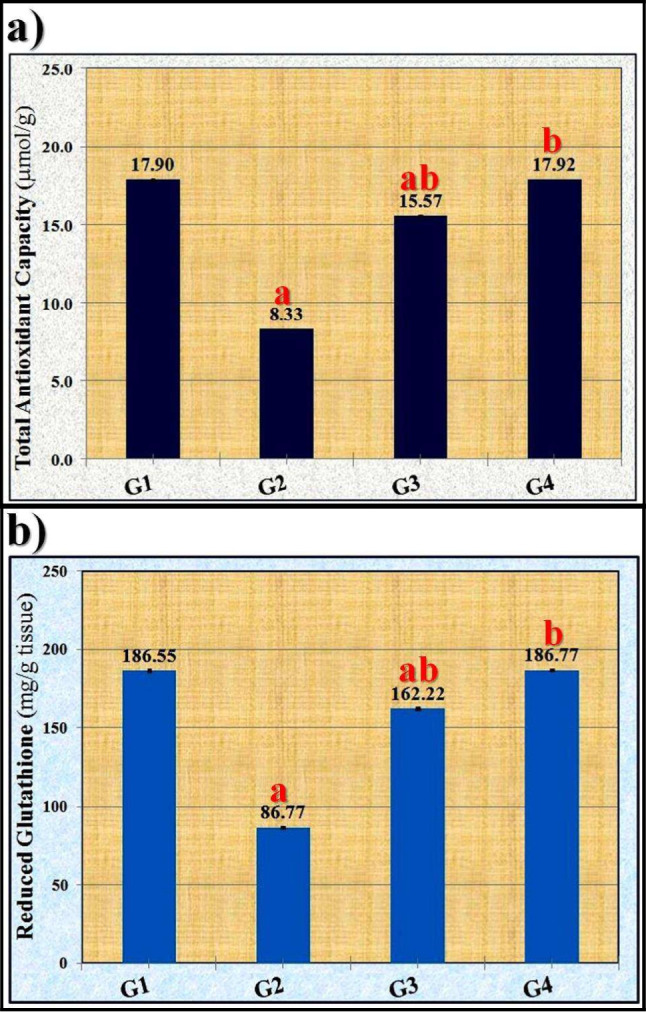
Effect of zinc sulfate (ZnSO_4_) against depletion of the antioxidant system induced by olanzapine on **a** total antioxidant capacity (TAC) and **b** concentration of reduced glutathione (GSH) in liver tissue of rats. The data were statistically examined using the Bonferroni test as a post-hoc analysis. “a” denotes significant difference (*p* ≤ 0.05) compared to control group, “b” denotes significant difference (*p* ≤ 0.05) compared to toxic group. G1: control group, G2: OLZ-treated group, G3: OLZ + ZnSO_4_ at a dose of 50 mg/kg treated group, and G4: OLZ + ZnSO_4_ at a dose of 100 mg/kg treated group

TAC and GSH levels in the liver tissue homogenates were significantly (*p* ≤ 0.05) reduced by OLZ, according to the results in Fig. 1. These values increased significantly (*p* ≤ 0.05) in the tissue of the zinc sulfate (ZnSO_4_)-treated groups in a dose-dependent manner when compared to the OLZ-treated group. These values reverted to normal after receiving a dose of 100 mg/kg of ZnSO_4_.

**Fig. 2:**
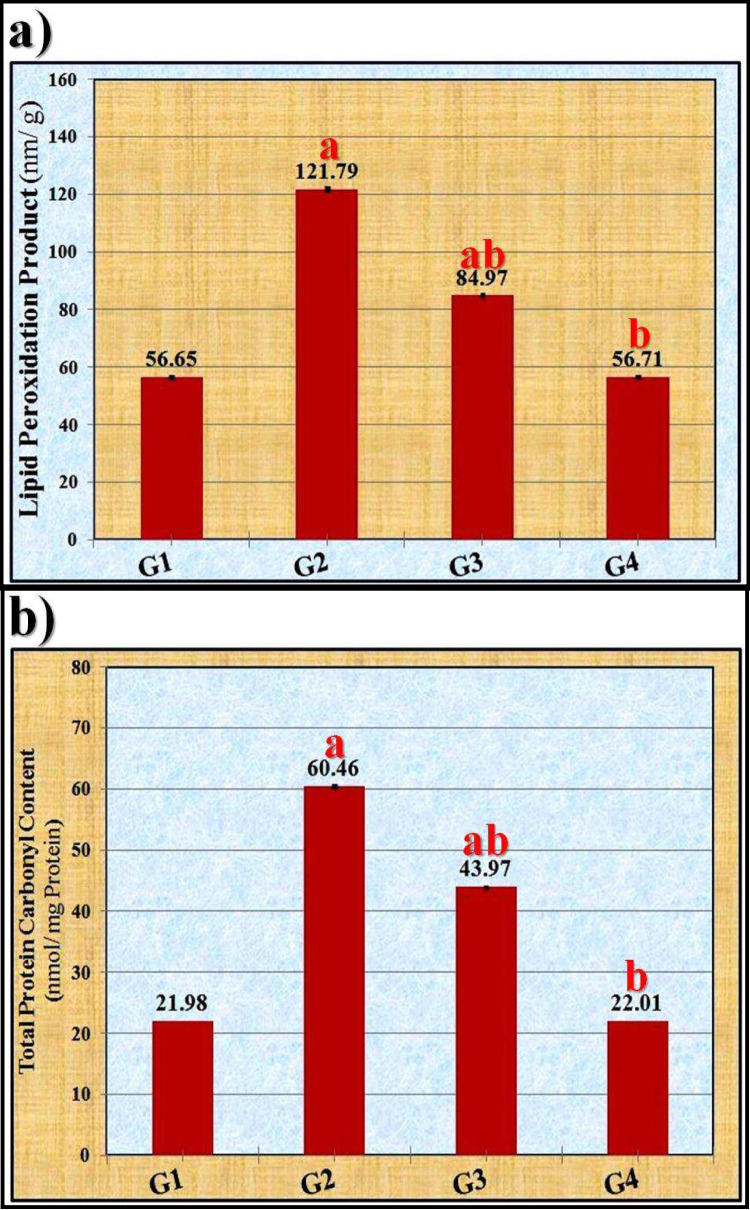
Effect of zinc sulfate (ZnSO_4_) against the oxidative stress induced by olanzapine on **a** lipid peroxidation products (LPO) and **b** total protein carbonyl content (TPC) in liver tissue of rats. The data were statistically examined using the Bonferroni test as a post-hoc analysis. “a” denotes significant difference (*p* ≤ 0.05) compared to the control group, “b” denotes significant difference (*p* ≤ 0.05) compared to the toxic group. G1: control group, G2: OLZ-treated group, G3: OLZ + ZnSO_4_ at a dose of 50 mg/kg treated group, and G4: OLZ + ZnSO_4_ at a dose of 100 mg/kg treated group

Rats treated with OLZ had significantly (*p* ≤ 0.05) higher levels of LPO and TPC than the control group in terms of the products of peroxidation reactions (Fig. 2). The concentrations of these indicators were significantly (*p* ≤ 0.05) reduced in the ZnSO_4_-treated groups at both doses (50 and 100 mg/kg) in a dose-dependent manner. Rats given 100 mg/kg of ZnSO_4_ showed a recovery to normal values in these assays in their livers.

### Inflammatory and Fibrotic Markers

Indicators of tissue integrity include levels of inflammatory markers (TNF-α and IL-6) and fibrotic marker (hydroxyproline). Rats treated with OLZ showed significantly (*p* ≤ 0.05) higher levels of the inflammatory markers TNF-α and IL-6 than the control group (Table 1). At both doses (50 and 100 mg/kg), ZnSO_4_ administration significantly (*p* ≤ 0.05) reduced these markers’ levels in the ZnSO_4_-treated groups in a dose-dependent manner. Rats given 100 mg/kg of ZnSO_4_ had their liver levels of these indicators returned to normal. The group that received OLZ orally showed no change in the amount of the fibrotic marker, hydroxyproline. ZnSO_4_ administration at the two doses under study did not alter this marker’s level.

**Table 1:** Effect of zinc sulfate (ZnSO_4_) against the alterations in markers of the inflammatory response induced by olanzapine (OLZ) in the liver tissues of rats

	G1	G2	G3	G4
TNF-α (pg/g tissue)	229.68 ± 2.84	401.93 ± 4.97^**a**^	321.55 ± 3.97^**ab**^	232.33 ± 2.87^**b**^
IL-6 (pg/g tissue)	287.10 ± 3.55	502.42 ± 6.21^**a**^	401.93 ± 4.97^**ab**^	290.41 ± 3.59^**b**^
Hydroxyproline (µg/mg tissue)	0.52 ± 0.01	0.55 ± 0.01	0.54 ± 0.01	0.58 ± 0.01

Data were presented as mean ± SE (from five replicates). The data were statistically examined using the Bonferroni test as a post-hoc analysis. “**a**” denotes significant difference (*p* ≤ 0.05) compared to the control group and “**b**” denotes significant difference (*p* ≤ 0.05) compared to the toxic group. G1: control group, G2: OLZ-treated group, G3: OLZ + ZnSO_4_ at a dose of 50 mg/kg treated group, and G4: OLZ + ZnSO_4_ at a dose of 100 mg/kg treated group

### Histopathological Examination

**Fig. 3:**
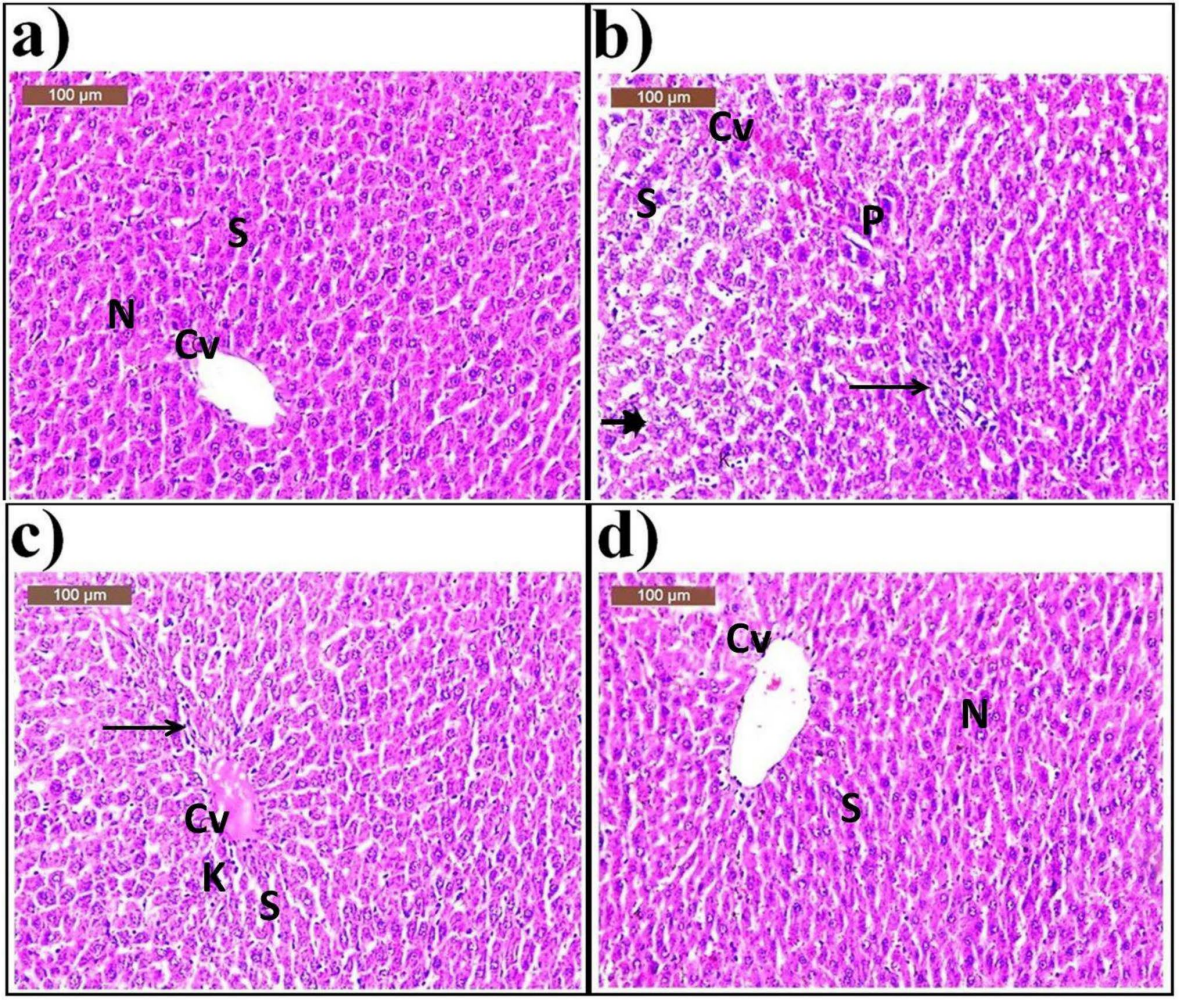
A photomicrograph of rat liver of **a** control group (G1) showing normal hepatic architecture, central vein (Cv), blood sinusoids (S), and nucleus (N); **b** OLZ-treated group (G2) showing dilated congested central veins (Cv), necrosis (arrowhead), with pyknotic nuclei (P) and increase in kupffer cells (K). Notice a focal area of aggregations of lymphocytes infiltrating (star) in between the hepatocytes; **c** OLZ + ZnSO_4_ at a dose of 50 mg/kg treated group (G3) showing moderate improvement as congestion of central vein (Cv), minimal inflammatory cells (arrow) with slight dilated blood sinusoids (S), and mild increase in kupffer cells (K); and **d** OLZ + ZnSO_4_ at a dose of 100 mg/kg treated group (G4) showing remarkable improvement of histological structure, normal appearance of central vein (Cv), and nucleus (N) associated with slight dilated blood sinusoids (S)

The sections of the liver in the control group (G1) showed typical hepatic architecture, with hepatocytes distributed in cords extending from the central veins. Hepatocytes contained spherical vesicular nuclei with blood sinusoids (Fig. 3a).

In the OLZ-treated group (G2), the histopathological examination of hepatic tissues showed dilated congested central veins, necrosis with pyknotic nuclei, and an increase in Kupffer cells. There was a focal area of aggregations of lymphocytes infiltrating between the hepatocytes **(**Fig. 3b). In the rats treated with OLZ coinciding with ZnSO_4_ at a dose of 50 mg/kg (G3), the examination section showed moderate improvement such as congestion of the central vein, minimal inflammatory cells with slightly dilated blood sinusoids, and a mild increase in Kupffer cells (Fig. 3c). In the rats treated with OLZ coinciding with ZnSO_4_ at a dose of 100 mg/kg (G4), the sections of liver tissues showed a remarkable improvement in histological structure, normal appearance of the central vein and nucleus, and slightly dilated blood sinusoids (Fig. 3d).

### Electrophoretic Assays

#### Native Protein Patterns

##### Protein Pattern

**Fig. 4:**
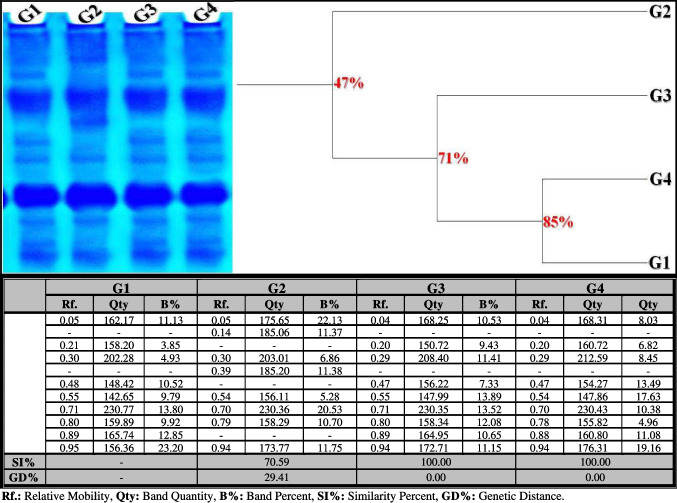
Native electrophoretic protein pattern showing the physiological effect of zinc sulfate (ZnSO_4_) against the toxicity induced by olanzapine on number and arrangement of the enzyme bands in liver tissue of rats. G1: control group, G2: OLZ treated group, G3: OLZ + ZnSO_4_ at a dose of 50 mg/kg treated group, and G4: OLZ + ZnSO_4_ at a dose of 100 mg/kg treated group. Rf., relative mobility; Qty, band quantity; B%, band percent; SI%, similarity percent; GD%, genetic distance

The native protein pattern showed nine bands (Rfs 0.05, 0.21, 0.30, 0.48, 0.55, 0.71, 0.80, 0.89, and 0.95; Qty 162.17, 158.20, 202.28, 148.42, 142.65, 230.77, 159.89, 165.74, and 156.36; B% 11.13, 3.85, 4.93, 10.52, 9.79, 13.80, 9.92, 12.85, and 23.20, respectively) in the control liver (Fig. 4). Six common bands (Rfs 0.05, 0.30, 0.55, 0.71, 0.80, and 0.95) were identified. In the OLZ-treated group, the physiological changes were represented by the disappearance of three normal bands and the appearance of two characteristic (abnormal) ones (Rfs 0.14 and 0.39; Qty 185.06 and 185.20; B% 11.37 and 11.38, respectively). Therefore, the SI value (SI = 70.59%; GD = 29.41%) decreased compared to the control group. In the rats treated with OLZ coinciding with ZnSO_4_ at both doses, the protein pattern was improved by hiding the abnormal ones while restoring the absent normal bands (Rfs 0.20, 0.47, and 0.89) in the ZnSO_4_-treated group at a dose of 50 mg/kg (Qty 150.22, 156.22, and 164.95; B% 9.43, 7.33, and 10.65, respectively) and in the ZnSO_4_-treated group at a dose of 100 mg/kg (Qty 160.72, 154.27, and 160.80; B% 6.82, 13.49, and 11.08, respectively). Therefore, both treated groups are physiologically resembled to the control group by 100.00% (GD = 0.00%).

##### Lipid Moiety of Native Protein Pattern

**Fig. 5:**
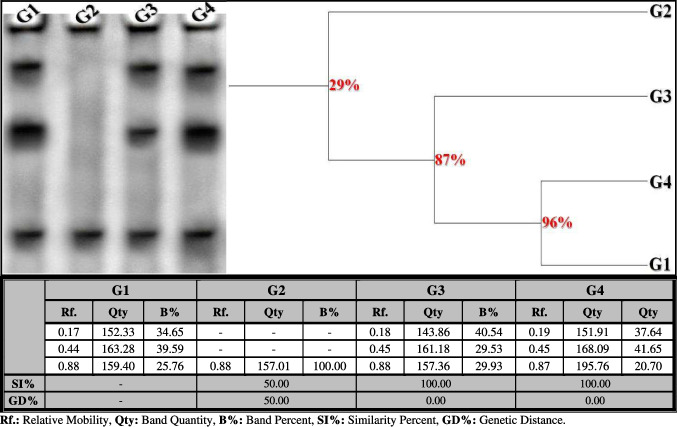
Electrophoretic lipid moiety of native protein pattern showing the physiological effect of zinc sulfate (ZnSO_4_) against the toxicity induced by olanzapine on number and arrangement of the enzyme bands in liver tissue of rats. G1: control group, G2: OLZ-treated group, G3: OLZ + ZnSO_4_ at a dose of 50 mg/kg treated group, and G4: OLZ + ZnSO_4_ at a dose of 100 mg/kg treated group. Rf., relative mobility; Qty, band quantity; B%, band percent; SI%, similarity percent; GD%, genetic distance

The native pattern showed three bands (Rfs 0.17, 0.44, and 0.88; Qty 152.33, 163.28, and 159.40; B% 34.65, 39.59, and 25.76, respectively) in the control liver (Fig. 5). Only one common band (Rf 159.40) was identified. In the OLZ-treated group, the physiological changes were represented by the disappearance of two normal bands without the appearance of abnormal ones. Therefore, the SI value (SI = 50.00%; GD = 50.00%) decreased compared to the control group. In the rats treated with OLZ coinciding with ZnSO_4_ at both doses, the native pattern was improved by restoring the absent normal bands (Rfs 0.17 and 0.44) in the ZnSO_4_-treated group at a dose of 50 mg/kg (Qty 143.86 and 161.18; B% 40.54 and 29.53, respectively) and in the ZnSO_4_-treated group at a dose of 100 mg/kg (Qty 151.91 and 168.09; B% 37.64 and 41.65, respectively). Therefore, at the physiological state, both treated groups are completely similar to the control group (SI = 100.00%; GD = 0.00%).

##### Calcium Moiety of Native Protein Pattern

**Fig. 6:**
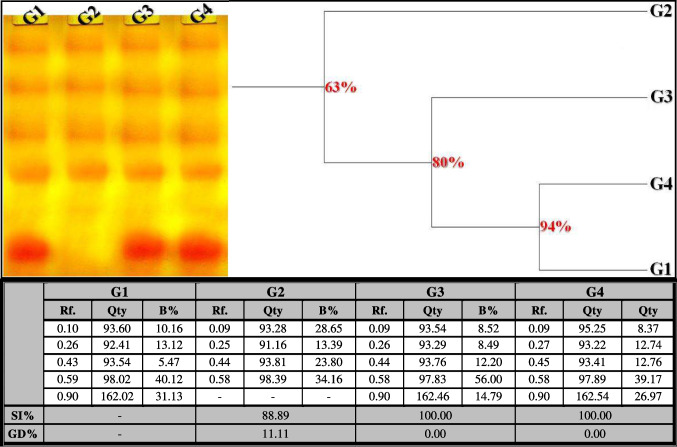
Electrophoretic calcium moiety of native protein pattern showing the physiological effect of zinc sulfate (ZnSO_4_) against the toxicity induced by olanzapine on number and arrangement of the enzyme bands in liver tissue of rats. G1: control group, G2: OLZ-treated group, G3: OLZ + ZnSO_4_ at a dose of 50 mg/kg treated group, and G4: OLZ + ZnSO_4_ at a dose of 100 mg/kg treated group. Rf., relative mobility; Qty, band quantity; B%, band percent; SI%, similarity percent; GD%, genetic distance

In the control liver, the native protein pattern showed five bands (Rfs 0.10, 0.26, 0.43, 0.59, and 0.90; Qty 93.60, 92.41, 93.54, 98.02, and 162.02; B% 10.16, 13.12, 5.47, 40.12, and 31.13, respectively) (Fig. 6). Four common bands (Rfs 0.10, 0.26, 0.43, and 0.59) were identified. No unique (characteristic) bands were detected. The physiological changes caused by OLZ injection were represented by the disappearance of one normal band without the appearance of abnormal ones. Therefore, the physiological similarity to the control group decreased to 88.89% (GD = 11.11%). In the rats treated with OLZ coinciding with ZnSO_4_ at both doses, the native pattern was improved by restoring the previously absent normal band (Rf 0.90) in the ZnSO_4_-treated group at a dose of 50 mg/kg (Qty 162.46; B% 14.79) and in the ZnSO_4_-treated group at a dose of 100 mg/kg (Qty 162.54; B% 26.97). Therefore, at the physiological state, both treated groups are identical to the control group by 100.00% (GD = 0.00%).

#### Native Isoenzyme Patterns

##### Catalase (CAT) Pattern

**Fig. 7:**
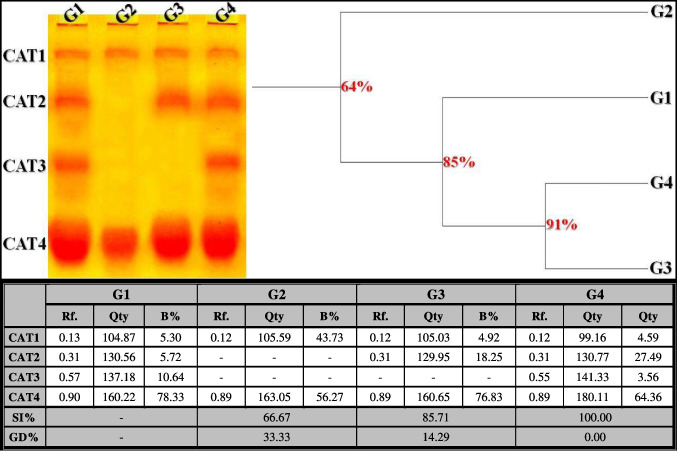
Electrophoretic catalase (CAT) isoenzyme pattern showing the physiological effect of zinc sulfate (ZnSO_4_) against the toxicity induced by olanzapine on the number and arrangement of the enzyme bands in liver tissue of rats. G1: control group, G2: OLZ-treated group, G3: OLZ + ZnSO_4_ at a dose of 50 mg/kg treated group, and G4: OLZ + ZnSO_4_ at a dose of 100 mg/kg treated group. Rf., relative mobility; Qty, band quantity; B%, band percent; SI%, similarity percent; GD%, genetic distance

The CAT isoenzyme pattern in the control liver tissue was represented by four types (Rfs 0.13, 0.31, 0.57, and 0.90; Qty 104.87, 130.56, 137.18, and 160.22; B% 5.30, 5.72, 10.64, and 78.33, respectively) (Fig. 7). Two common bands were identified (CAT1 and CAT4; Rfs 0.13 and 0.90, respectively). The quantitative abnormalities caused by the treatment with OLZ were represented by the disappearance of two CAT types (CAT2 and CAT3). The physiological similarity of this group to the control group decreased to 66.67% (GD = 33.33%). The isoenzyme pattern was significantly improved by reintroducing only one missing normal (CAT2) type (Rf 0.31; Qty 129.95; B% 18.25) in the rats that received ZnSO_4_ treatment at a dose of 50 mg/kg. This closely resembled the control group at a physiological level by 85.71% (GD = 14.29%). The deleterious effect induced by OLZ was alleviated by restoring the two absent CAT types (CAT2 and CAT3) identified at Rfs 0.31 and 0.55 (Qty 130.77 and 141.33; B% 27.49 and 3.56, respectively) in the rats that received ZnSO_4_ treatment at a dose of 100 mg/kg. This completely resembled the control group at a physiological level (SI = 100.00%; GD = 0.00%).

##### Peroxidase (POX) Pattern

**Fig. 8:**
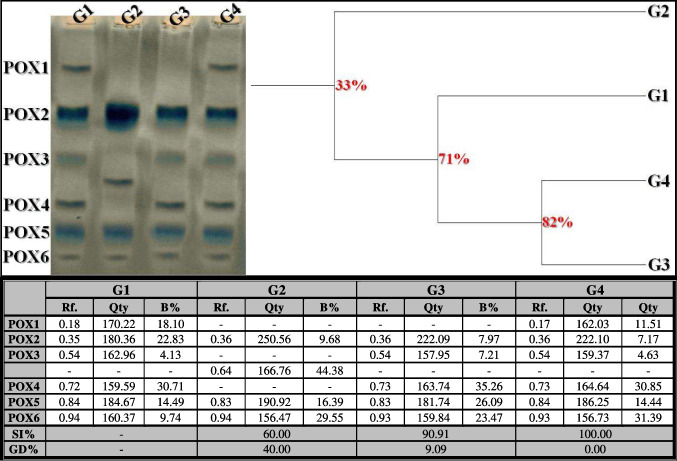
Electrophoretic peroxidase (POX) isoenzyme pattern showing the physiological effect of zinc sulfate (ZnSO_4_) against the toxicity induced by olanzapine on number and arrangement of the enzyme bands in liver tissue of rats. G1: control group, G2: OLZ-treated group, G3: OLZ + ZnSO_4_ at a dose of 50 mg/kg treated group, and G4: OLZ + ZnSO_4_ at a dose of 100 mg/kg treated group. Rf., relative mobility; Qty, band quantity; B%, band percent; SI%, similarity percent; GD%, genetic distance

In the control liver tissue, the POX isoenzyme pattern was represented by six types (Rfs 0.18, 0.35, 0.54, 0.72, 0.84, and 0.94; Qty 170.22, 180.36, 162.96, 159.59, 184.67, and 160.37; B% 18.10, 22.83, 4.13, 30.71, 14.49, and 9.74, respectively) (Fig. 8). Three common bands were identified (POX2, POX5, and POX6; Rfs 0.35, 0.84, and 0.94, respectively). In the OLZ-injected group, the disappearance of three normal POX types (POX1, POX3, and POX4) and the appearance of one characteristic abnormal band (Rf 0.64; Qty 166.76; B% 44.38) were indicative of qualitative changes. The physiological similarity of this group to the control group decreased to 60.00% (GD = 40.00%). The isoenzyme pattern was significantly improved by the disappearance of the characteristic band with the reintroduction of only two missing normal types (POX3 and POX4) (Rfs 0.54 and 0.73; Qty 157.95 and 163.74; B% 7.21 and 35.26, respectively) in the rats that received ZnSO_4_ treatment at a dose of 50 mg/kg. This closely resembled the control group at a physiological level by 90.91% (GD = 9.09%). The abnormalities caused by OLZ were alleviated by the disappearance of the characteristic band with the restoration of the three absent POX types (POX1, POX3, and POX4) identified at Rfs 0.17, 0.54, and 0.73 (Qty 162.03, 159.37, and 164.64; B% 11.51, 4.63, and 30.85, respectively) in the rats that received ZnSO_4_ treatment at a dose of 100 mg/kg. This was completely similar to the control group at the qualitative level (SI = 100.00%; GD = 0.00%).

##### α-Amylase (α-Amy) Pattern

**Fig. 9:**
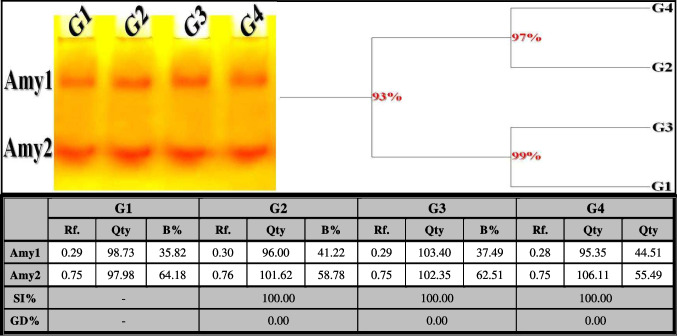
Electrophoretic amylase (Amy) isoenzyme pattern showing the physiological effect of zinc sulfate (ZnSO_4_) against the toxicity induced by olanzapine on number and arrangement of the enzyme bands in liver tissue of rats. G1: control group, G2: OLZ-treated group, G3: OLZ + ZnSO_4_ at a dose of 50 mg/kg treated group, and G4: OLZ + ZnSO_4_ at a dose of 100 mg/kg treated group. Rf., relative mobility; Qty, band quantity; B%, band percent; SI%, similarity percent; GD%, genetic distance

In the control liver tissue, this isoenzyme pattern appeared as two types (Rfs 0.29 and 0.75; Qty 98.73 and 97.98; B% 35.82 and 64.18, respectively) and was considered common bands (Fig. 9). No physiological or quantitative changes were detected in the OLZ-injected group. The treatment with ZnSO_4_ at both studied doses (50 and 100 mg/kg) showed no electrophoretic changes affecting the physiological similarity compared to the control group (SI = 100.00%; GD = 0.00%).

##### β-Esterase (β-EST) Pattern

**Fig. 10:**
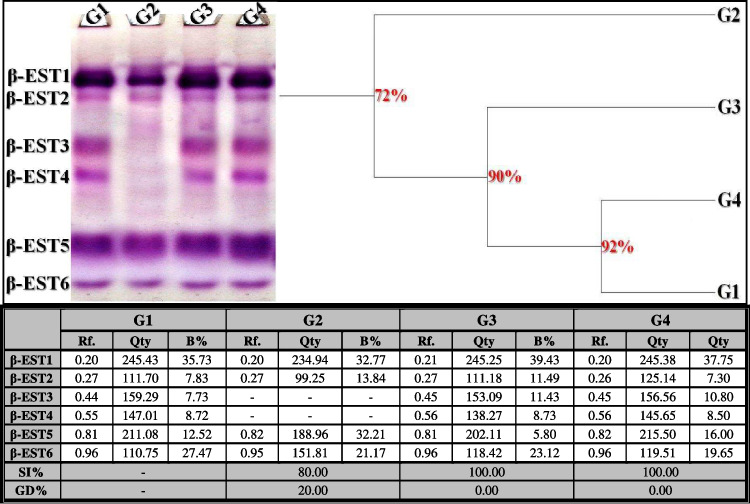
Electrophoretic β-esterase (β-EST) isoenzyme pattern showing the physiological effect of zinc sulfate (ZnSO_4_) against the toxicity induced by olanzapine on number and arrangement of the enzyme bands in liver tissue of rats. G1: control group, G2: OLZ-treated group, G3: OLZ + ZnSO_4_ at a dose of 50 mg/kg treated group, and G4: OLZ + ZnSO_4_ at a dose of 100 mg/kg treated group. Rf., relative mobility; Qty, band quantity; B%, band percent; SI%, similarity percent; GD%, genetic distance

In the control liver tissue, the β-EST isoenzyme pattern was represented by six types (Rfs 0.20, 0.27, 0.44, 0.55, 0.81, and 0.96; Qty 245.43, 111.70, 159.29, 147.01, 211.08, and 110.75; B% 35.73, 7.83, 7.73, 8.72, 12.52, and 27.47, respectively) (Fig. 10). Four common bands were identified (β-EST1, β-EST2, β-EST5, and β-EST6; Rfs 0.20, 0.27, 0.81, and 0.96, respectively). In the OLZ-injected group, the physiological alterations were represented by the disappearance of two normal types (β-EST3 and β-EST4). As a result, this group showed the lowest physiological similarity to the control group (SI = 80.00%; GD = 2.00%). In the rats treated with OLZ coinciding with ZnSO_4_ at both doses, the isoenzyme pattern was improved by restoring the previously absent normal types (β-EST3 and β-EST4; Rf 0.45 and 0.56, respectively) in the ZnSO_4_-treated group at a dose of 50 mg/kg (Qty 153.09 and 138.27; B% 11.43 and 8.73, respectively) and in the ZnSO_4_-treated group at a dose of 100 mg/kg (Qty 156.56 and 145.65; B% 10.80 and 8.50, respectively). Therefore, in the physiological state, both treated groups resembled the control group by 100.00% (GD = 0.00%).

### Absorption Spectra of Hemoglobin

**Fig. 11:**
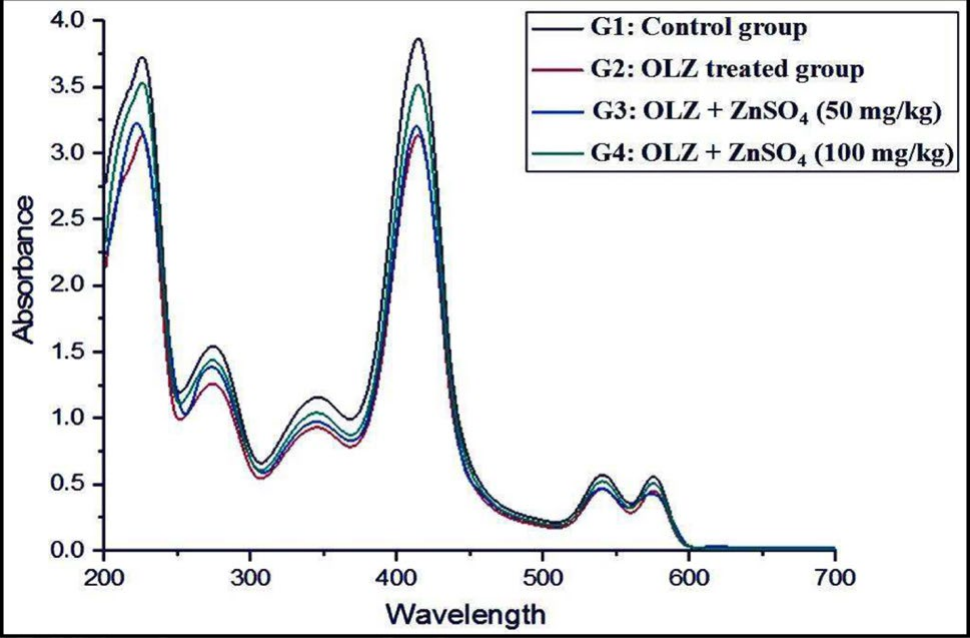
Hemoglobin absorption spectra of treated groups compared to the control group. G1: control group, G2: OLZ-treated group, G3: OLZ + ZnSO_4_ at a dose of 50 mg/kg treated group, and G4: OLZ + ZnSO_4_ at a dose of 100 mg/kg treated group

Table 2 and Fig. 11 show the absorption spectra and associated absorbance bands of hemoglobin for all treatment groups as compared to the control group. During the current experiment, the concentration of hemoglobin for all groups under these conditions was found to be 3.4 × 10^−5^ M. The absorbance band at 340 nm (indicating globin-heme interaction) decreased significantly in the OLZ-treated group (G2), but groups treated with ZnSO_4_ (50 or 100 mg/kg) showed a considerable increase in absorbance at the same wavelength compared to the control group. Furthermore, animals treated with OLZ in combination with ZnSO_4_ at a dosage of 100 mg/kg (G4) had a significantly higher hemoglobin Soret band at 415 nm than rats given OLZ alone (G2). The absorbance band at 540 nm fell considerably in the groups treated with various levels of ZnSO_4_; although, a significant rise was seen in the group treated with OLZ in combination with ZnSO_4_ at a dosage of 50 mg/kg (G3) compared to the OLZ-treated group (G2). In rats treated with OLZ plus ZnSO_4_ at a dosage of 100 mg/kg (G4), the absorbance band at 578 nm (indicating heme-heme interaction) increased significantly compared to the OLZ-treated group (G2). The absorbance ratio A578/A540 was less than one in all groups, with a significant drop in the OLZ-treated group (G2) and a significant rise in the groups treated with ZnSO_4_ at all dosages (50 and 100 mg/kg) compared to the control group.

**Table 2:** Absorbance of different hemoglobin bands of all treated groups compared to the control group

Group	Globin-heme interaction (*λ* = 340 nm)	Soret band (*λ* = 415 nm)	*λ* = 540 nm	Heme-heme interaction (*λ* = 578 nm)	Ratio A578/A540
G_1_	0.7315 ± 0.0024	1.1725 ± 0.0167	0.691 ± 0.00173	0.642 ± 0.00056	0.9290
G_2_	0.5471 ± 0.00271^a^	1.1367 ± 0.038^a^	0.543 ± 0.0022^a^	0.423 ± 0.00034^a^	0.7790
G_3_	0.7030 ± 0.0025^ab^	1.1531 ± 0.202^ab^	0.679 ± 0.00062^ab^	0.579 ± 0.00046^ab^	0.8527
G_4_	0.7164 ± 0.0028^ab^	1.1631 ± 0.215^ab^	0.685 ± 0.00051^ab^	0.599 ± 0.00043^ab^	0.8744

Data were presented as mean ± SE (from five replicates). The data were statistically examined using the Bonferroni test as a post-hoc analysis. “**a**” denotes significant difference (*p* ≤ 0.05) compared to the control group and “**b**” denotes significant difference (*p* ≤ 0.05) compared to the toxic group. G1: control group, G2: OLZ-treated group, G3: OLZ + ZnSO_4_ at a dose of 50 mg/kg treated group, and G4: OLZ + ZnSO_4_ at a dose of 100 mg/kg treated group

## Discussion

OLZ may have adverse consequences including weight gain, elevated plasma lipid levels, and hormonal abnormalities, despite being often used to treat schizophrenia and other psychiatric diseases [33]. According to the results of the current investigation, OLZ raised the levels of peroxidation products (LPO and TPC) by decreasing antioxidant system markers (TAC and GSH). This supports the theory of Miljevic et al. [34] that OLZ therapy causes oxidative stress, which could be a mechanism by which it raises the risk of diabetes and metabolic syndrome and causes these distinct unfavorable metabolic consequences. According to Xu and Zhuang [35], oxidative cell damage also leads to an increase in the oxidation of proteins and lipids after OLZ therapy. Furthermore, according to Holt [36], the activation of superoxide production—which is connected to the depletion of GSH—increases the level of malondialdehyde, an indicator of lipid peroxides, in the liver tissues of rats treated with OLZ. The administration of OLZ has been shown by Raben et al. [37] to enhance oxidative stress and cell damage in certain organs, particularly the liver, as a result of an increase in reactive oxygen species (ROS) generation. Supplementation with ZnSO_4_ increases the antioxidant state of the liver tissues, which is similar to the findings of Mard et al. [38], who showed that Zn helps reduce ROS production while preserving tissue integrity and function by stabilizing the activity of antioxidant enzymes. Zn also reduces collagen accumulation in the tissue and has an important physiological role in controlling cell shape and function.

TNF-α and IL-6 levels in the liver of the rats that received OLZ were significantly elevated in terms of inflammatory markers. This is consistent with the results of Calevro et al. [39] and Mahmoud and El-Deek [40], who pointed out that OLZ increased the pro-inflammatory cytokines IL-6 and TNF-α, which might have important consequences for metabolic dysregulation. Increases in TNF-α and IL-6 mRNA expression levels may raise the levels of these indicators [41]. When Zn was administered in addition to OLZ, the mean levels of these cytokines were considerably lower. The findings of Bonaventura et al. [42], who demonstrated that Zn preparations can reduce the production of TNF-α and IL-6 among other inflammatory cytokines, are consistent with this finding. According to Ni et al. [43], zinc preparations (ZnSO_4_) may have an anti-inflammatory impact because of their antioxidant function, which stabilizes cell membranes and lowers apoptosis.

The histopathological analysis demonstrated severe damage to the liver tissue as a result of OLZ treatment. This is consistent with the findings of Abd El-Hameed et al. [2], who observed that the liver of the OLZ-treated group had increased portal area congestion, severe lipid alterations, inflammatory infiltration, and vacuolization of hepatocytes. These effects could be linked to the production of ROS, which damage various cell membrane components. According to Contreras-Zentella and Hernández-Muñoz [44], these alterations are linked to increased lipid peroxidation, which causes cellular architecture to degenerate or be destroyed. Furthermore, the release of reactive species, cytokines, and chemokines from Kupffer cells, which led to the stimulation of extravasation and activation of neutrophils, explained the cellular infiltration [45]. The liver histoarchitecture was improved and restored to its typical state upon treatment with ZnSO_4_ at a dose of 100 mg/kg. This might be related to the stimulated antioxidant defense system in the liver in the presence of Zn, which reduces tissue injury caused by a free radical–mediated mechanism [46]. Moreover, ZnSO_4_ administration stimulates the synthesis of MT, which is responsible for maintaining the integrity of organelle membranes by increasing the expression of its mRNAs [47].

Physiological differences between treated groups can be detected using the electrophoretic method [48]. To ensure that various proteins and isoenzymes can carry out their biological roles, they are employed to evaluate their integrity [49]. By employing the SI percentage values as an analytical tool in conjunction with electrophoretic methods, the physiological condition of the tissue may be observed. High SI values indicate that the two samples’ band counts and configurations are almost the same [50].

The results of this study demonstrated that the OLZ-treated group had an altered native protein pattern. These changes might be linked to metabolic issues, which result in a reduction of glutathione and antioxidant activities as well as an excess of ROS [51]. Furthermore, by altering the way proteins fold, the excess ROS make it more difficult for chaperones to carry out their biological role [52].

An increase in ROS that target the lipid portion and oxidatively modify the lipid moiety of proteins may be the source of the physiological changes brought on by OLZ in the lipid moiety of the native protein pattern [53]. The protein binds to lipoproteins, of course. Consequently, modifying the lipid moiety may result in a change in the protein pattern [54].

Because OLZ inhibits the mineralization process, which is necessary for tissue protection and detoxification against harmful substances, it may alter the calcium moiety of the native protein pattern [55]. Variations in the tissue’s calcium ion concentration may modify this natural cycle [56].

Extracellular Zn is effective in enhancing insulin metabolism and lowering blood glucose levels through regulation of the insulin-signaling pathway or interaction with insulin receptors. Administering ZnSO_4_ improved the protein pattern and returned it to its normal physiological state [57].

Zn administration prevented oxidative damage to lipids and proteins in the tissue under study, leading to an increase in GSH levels. This, in turn, reduced peroxidation and carbonylation processes [58]. Furthermore, the efficiency of Zn in preventing oxidative stress resulting from an imbalance in oxidative/antioxidative processes helps protect cellular macromolecules from damage [59].

Antioxidant enzymes are tissue-dependent tools because their activity varies depending on the tissue. The electrophoretic patterns of CAT and POX in the liver tissues under study were changed by OLZ treatment. De Freitas et al. [60] claim that this shift may be connected to oxidative stress, altered metabolic pathways, and the generation of too many ROS, which might result in the breakdown of protein content and affect physicochemical and immunologic traits. The changed electrophoretic isoenzyme pattern may be a sign of a shift in the rate of protein production brought on by ROS-induced DNA damage, which would affect the fractional activities of the proteins [61]. Furthermore, elevated glucose levels may be a factor in these electrophoretic alterations by glycating these enzymes [62].

Since EST enzymes may accelerate the breaking of ester linkages to release acetylcholine during nervous system stimulation, it is widely accepted that they play an important role in the neurotransmission process [63]. Additionally, they hydrolyze neutral lipids that are delivered into cells into the appropriate carboxylic acids, which is necessary for their hypolipidemic action [64].

According to the current study, the differences in the electrophoretic β-EST pattern in the liver of the group treated with OLZ may be caused by changes in hormones, metabolism, or the expression of the protein part responsible for its enzymatic activity [65]. According to Seif et al. [63], deviations in the β-EST pattern might be caused by ROS’s impact on the protein molecule’s integrity, which could vary the pace at which reactive species-induced protein production occurs and change fractional activity linked to DNA damage. Protein instability and disintegration might result from glycosylation of β-EST types brought on by elevated glucose levels from the OLZ-induced metabolic disease [66].

The physiological abnormalities in all electrophoretic isoenzyme (CAT, POX, and β-EST) patterns that were caused in the liver of rats treated with OLZ were alleviated in a dose-dependent manner by administering the ZnSO_4_ solution. This is due to the effectiveness of exogenous Zn in boosting antioxidant status and improving antioxidant defense against ROS and peroxidation processes, protecting these vital biomacromolecules from oxidation [67, 68]. The MT, which is stimulated by Zn and contains cysteine residues, plays a significant role in reducing oxidative stress. MT overexpression in metabolic organs effectively reduces oxidative damage caused by hyperglycemia [69]. As a result, it reduces the electrophoretic changes caused by the glycosylation process.

Hemoglobin’s absorption spectrum can reveal important information about structural changes in the biomolecule. Reduced absorption at 340 nm and decreased Soret band absorbance (415 nm) in the OLZ-treated group relative to the control group indicate that hemoglobin’s structure has changed as a result of oxidative stress induced by OLZ. Through the activation of pro-inflammatory pathways, this oxidative stress likely contributes to cellular damage. For example, it has been demonstrated that OLZ activates nuclear factor kappa B (NF-κB), a transcription factor essential for inflammatory reactions. Tissue damage and metabolic dysfunction are exacerbated by the interaction of inflammation and oxidative stress. Increased ROS production, mitochondrial dysfunction, lipid peroxidation, and a compromised antioxidant defense system are the main causes of oxidative stress brought on by OLZ. Long-term usage of this imbalance may contribute to neurotoxicity and cause a number of adverse consequences, including metabolic diseases such as insulin resistance and dyslipidemia [70–73].

Hemoglobin stability during folding processes under stress is suggested by the notable rise in the Soret band absorbance at 415 nm and 340 nm in the rats treated with OLZ concurrently with ZnSO_4_ at a dosage of 100 mg/kg. In these conditions, hemoproteins showed increased binding to novel prosthetic groups, suggesting a change in globin-heme interactions [74].

The instability of oxyhemoglobin as a macromolecule is reflected in the OLZ-treated group’s drop in absorbance at 578 nm (heme-heme interaction) and the accompanying fall in the A578/A540 ratio. However, the enhanced heme-heme interaction and increase in the A578/A540 ratio showed that these alterations were reversed after ZnSO_4_ treatment. This implies that Met-Hb is reduced to HbO_2_, improving hemoglobin’s ability to carry oxygen and stabilizing oxyhemoglobin [75, 76].

By increasing the activity of enzymes such as superoxide dismutase (SOD), preserving glutathione levels, and lowering lipid peroxidation, Zn sulfate functions as a potent antioxidant. It is a potential drug for lowering oxidative stress and metabolic dysfunction brought on by OLZ because of its capacity to reduce inflammation, safeguard mitochondrial function, and enhance insulin sensitivity. For patients receiving long-term OLZ treatment, Zn supplementation may help reduce OLZ-induced adverse effects and enhance general health [77, 78].

## Conclusion

Our findings reveal that Zn treatment improved the dynamic motion of hemoglobin and enhanced oxygen utilization. The administration of Zn along with OLZ may be favorable for schizophrenic patients since it reduced its adverse side effects. Moreover, the changes induced by OLZ in the antioxidants and inflammatory markers in the liver tissues, in addition to the severe lesions that were detected histopathologically, were alleviated by the ZnSO_4_ salt solution administered in a dose-dependent manner. Levels of these markers were restored to normal values by the highest ZnSO_4_ dose (100 mg/kg). Furthermore, the alterations caused by OLZ in native protein, lipid, and calcium moieties of the electrophoretically identified protein patterns were diminished by the ZnSO_4_ salt solution at both studied doses. Regarding the isoenzyme patterns, ZnSO_4_ normalized the physiological state of the electrophoretic CAT, POX, and β-EST isoenzyme patterns when administered at a dose of 100 mg/kg.

## Supplementary Information

Below is the link to the electronic supplementary material.

ESM 1 (DOC 2.01 MB)

## Data Availability

No datasets were generated or analysed during the current study.

## References

[1] Jennings AA, Guerin N, Foley T (2018) Development of a tool for monitoring the prescribing of antipsychotic medications to people with dementia in general practice: a modified eDelphi consensus study. Clin Interv Aging 13:2107–2117 30425465 10.2147/CIA.S178216 PMC6203170

[2] Abd El-Hameed AM, Eskandrani AA, Elroby FA (2020) Assessment of the ameliorative effect of *Hypericum perforatum* on olanzapine-induced hepatic oxidative stress and metabolic abnormalities in experimental male albino rats. J Taibah Univ Sci 14(1):1496–1502

[3] Grajales D, Vitor Ferreira V, Valverde AM (2019) Second-generation antipsychotics and dysregulation of glucose metabolism: beyond weight gain. Cells 8:1336–1363 31671770 10.3390/cells8111336 PMC6912706

[4] Freyberg Z, Aslanoglou D, Shah R, Ballon JS (2017) Intrinsic and antipsychotic drug-induced metabolic dysfunction in schizophrenia. Front Neurosci 11:432–445 28804444 10.3389/fnins.2017.00432 PMC5532378

[5] Chen X, Liu L, Zeng Y, Li D, Liu X, Hu C (2022) Olanzapine induces weight gain in offspring of prenatally exposed poly I: C rats by reducing brown fat thermogenic activity. Front Pharmacol 13:1001919 36249777 10.3389/fphar.2022.1001919 PMC9561095

[6] Monserrat-Mesquida M, Quetglas-Llabrés M, Capó X, Bouzas C, Mateos D, Pons A, Tur JA, Sureda A (2020) Metabolic syndrome is associated with oxidative stress and proinflammatory state. Antioxidants 9:236 32178436 10.3390/antiox9030236 PMC7139344

[7] Franco C, Sciatti E, Favero G, Bonomini F, Vizzardi E, Rezzani R (2022) Essential hypertension and oxidative stress: novel future perspectives. Int J Mol Sci 23:14489 36430967 10.3390/ijms232214489 PMC9692622

[8] Zhang S, Zhang Y, Peng N, Zhang H, Yao J, Li Z, Liu L (2014) Pharmacokinetics and biodistribution of zinc-enriched yeast in rats. Sci World J 2014:217142 10.1155/2014/217142 PMC4151581 25215316

[9] Prasad AS (2014) Zinc: an antioxidant and anti-inflammatory agent: role of zinc in degenerative disorders of aging. J Trace Elem Med Biol 28(4):364–371 25200490 10.1016/j.jtemb.2014.07.019

[10] Abo-Ghanema II, Elnasharty MA, Sadek KM, Abd Elrehim AM (2016) Effects of zinc supplementation on some hematological and biochemical parameters in liver injured rats. Alex J Vet Sci 50(1):115–121

[11] Gheitasi I, Doustimotlagh AH, Kokhdan EP, Akbari G, Barmak MJ (2023) Renoprotective effects of zinc sulfate against transient liver ischemia/reperfusion injury in rats. Heliyon 9(5):e15505 37153414 10.1016/j.heliyon.2023.e15505 PMC10160695

[12] Tunçdemir M, Ertürküner S, Özçelik D (2017) Investigation of lipid peroxidation and antiapoptotic effects of zinc aganist liver damage in diabetic rats. Hum Exp Toxicol 36(8):813–822 27609014 10.1177/0960327116666619

[13] Ardıç CM, Ilgın S, Baysal M, Karaduman AB, Kılıç V, Aydoğan-Kılıç G, Uçarcan Ş, Atlı-Eklioğlu Ö (2021) Olanzapine induced reproductive toxicity in male rats. Sci Rep 11:4739 33637793 10.1038/s41598-021-84235-4 PMC7910427

[14] Ebaid H, Bashandy SAE, Hassan I, Al-Tamimi J, Haredy SA, Imbabi T, Omara EA, Bashandy YS, Awad EM (2024) The preventive effect of zinc sulfate against olanzapine-induced testicular toxicity in male rats. Biol Trace Elem Res. 10.1007/s12011-024-04442-8 39653981 10.1007/s12011-024-04442-8 PMC12174200

[15] Beutler E, Duron O, Kelly BM (1963) Improved method for the determination of blood glutathione. J Lab Clin Med 61:882–890 13967893

[16] Koracevic D, Koracevic G, Djordjevic V, Andrejevic S, Cosic V (2001) Method for the measurement of antioxidant activity in human fluids. J Clin Pathol 54(5):356–361 11328833 10.1136/jcp.54.5.356 PMC1731414

[17] Levine RL, Williams JA, Stadtman ER, Shacter E (1994) Carbonyl assays for determination of oxidatively modified proteins. Methods Enzymol 233:346–357 8015469 10.1016/s0076-6879(94)33040-9

[18] Ohkawa H, Ohishi N, Yagi K (1979) Assay for lipid peroxides in animal tissues by thiobarbituric acid reaction. Anal Biochem 95:351–358 36810 10.1016/0003-2697(79)90738-3

[19] Reddy GK, Enwemeka CS (1996) A simplified method for the analysis of hydroxyproline in biological tissues. Clin Biochem 29:225–229 8740508 10.1016/0009-9120(96)00003-6

[20] March CJ, Mosley B, Larsen A, Cerretti DP, Braedt G, Price V, Gillis S, Henney CS, Kronheim SR, Grabstein K (1985) Cloning, sequence and expression of two distinct human interleukin-1 complementary DNAs. Nature 315:641–647 2989698 10.1038/315641a0

[21] Engelmann H, Novick D, Wallach D (1990) Two tumor necrosis factor-binding proteins purified from human urine. Evidence for immunological cross-reactivity with cell surface tumor necrosis factor receptors. J Biol Chem 265:1531–1563 2153136

[22] Suvarna SK, Layton C, Bancroft JD (2019) Bancroft’s theory and practice of histological techniques. Churchill Livingstone Elsevier, Oxford

[23] Bradford MM (1976) A rapid and sensitive method for the quantitation of microgram quantities of protein utilizing the principle of protein-dye binding. Anal Biochem 72(1–2):248–254 942051 10.1016/0003-2697(76)90527-3

[24] Darwesh OM, Moawad H, Barakat OS, Abd El-Rahim WM (2015) Bioremediation of textile reactive blue azo dye residues using nanobiotechnology approaches. Res J Pharm, Biol Chem Sci 6(1):1202–1211

[25] Subramaniam HN, Chaubal KA (1990) Evaluation of intracellular lipids by standardized staining with a Sudan black B fraction. J Biochem Biophys Methods 21(1):9–16 1698849 10.1016/0165-022x(90)90040-j

[26] Abd Elhalim SA, Sharada HM, Abulyazid I, Aboulthana WM, Abd Elhalim ST (2017) Ameliorative effect of carob pods extract (Ceratonia siliqua L.) against cyclophosphamide induced alterations in bone marrow and spleen of rats. J App Pharma Science 7(10):168–181

[27] Siciliano MJ, Shaw CR (1976) Separation and visualization of enzymes on gels. In: Smith I (ed) Chromatographic and electrophoretic techniques. Yearbook Medical Publ, Chicago, pp 185–209

[28] Rescigno A, Sanjust E, Montanari L, Sollai F, Soddu G, Rinaldi AC, Oliva S, Rinaldi A (1997) Detection of laccase, peroxidase, and polyphenol oxidase on a single polyacrylamide gel electrophoresis. Anal Lett 30(12):2211–2220

[29] Rammesmayer G, Praznik W (1992) Fast and sensitive simultaneous staining method of Q-enzyme, α-amylase, R-enzyme, phosphorylase and soluble starch synthase separated by starch: polyacrylamide gel electrophoresis. J Chromatogr 623(2):399–402

[30] Ahmad A, Maheshwari V, Ahmad A, Saleem R, Ahmad R (2012) Observation of esterase-like-albumin activity during N′-nitrosodimethyl amine induced hepatic fibrosis in a mammalian model. Maced J Med Sci 5(1):55–61

[31] Nei M, Li WS (1979) Mathematical model for studying genetic variation in terms of restriction endonuclease. Proc Natl Acad Sci USA 76:5269–5273 291943 10.1073/pnas.76.10.5269 PMC413122

[32] Trivelli LA, Ranney HM, Lai H-T (1971) Hemoglobin components in patients with diabetes mellitus. N Engl J Med 284:353–357 5539916 10.1056/NEJM197102182840703

[33] Ersland KM, Myrmel LS, Fjære E, Berge RK, Madsen L, Steen VM, Skrede S (2019) One-year treatment with olanzapine depot in female rats: metabolic effects. Int J Neuropsychopharmacol 22(5):358–369 30854556 10.1093/ijnp/pyz012 PMC6499254

[34] Miljevic C, Nikolic M, Nikolic-Kokic CA, Jones DR, Niketic V, Lecic-Tosevski D, Spasic MB (2010) Lipid status, antioxidant enzyme defence and haemoglobin content in the blood of long-term clozapine-treated schizophrenic patients. Prog Neuropsychopharmacol Biol Psychiatry 34(2):303–307 19962416 10.1016/j.pnpbp.2009.11.024

[35] Xu H, Zhuang X (2019) Atypical antipsychotics-induced metabolic syndrome and non-alcoholic fatty liver disease: a critical review. Neuropsychiatr Dis Treat 15:2087–2099 31413575 10.2147/NDT.S208061 PMC6659786

[36] Holt RG (2019) Association between antipsychotic medication use and diabetes. Curr Diab Rep 19(10):96–106 31478094 10.1007/s11892-019-1220-8 PMC6718373

[37] Raben AT, Marshe VS, Chintoh A, Gorbovskaya I, Müller DJ, Hahn MK (2018) The complex relationship between antipsychotic-induced weight gain and therapeutic benefits: a systematic review and implications for treatment. Front Neurosci 11:741 29403343 10.3389/fnins.2017.00741 PMC5786866

[38] Mard SA, Akbari G, Dianat M, Mansouri E (2017) Protective effects of crocin and zinc sulfate on hepatic ischemia-reperfusion injury in rats: a comparative experimental model study. Biomed Pharmacother 96:48–55 28963950 10.1016/j.biopha.2017.09.123

[39] Calevro A, Cotel M-C, Natesan S, Modo M, Vernon AC, Mondelli V (2018) Effects of chronic antipsychotic drug exposure on the expression of translocator protein and inflammatory markers in rat adipose tissue. Psychoneuroendocrinology 95:28–33 29793094 10.1016/j.psyneuen.2018.05.021

[40] Mahmoud GS, El-Deek HE (2019) Melatonin modulates inflammatory mediators and improves olanzapine-induced hepatic steatosis in rat model of schizophrenia. Int J Physiol, Pathophysiol Pharmacol 11(3):64–75 31333809 PMC6628014

[41] Li W, Huang X, Deng C, Zhang B, Qian K, He M, Sun T (2021) Olanzapine induces inflammation and immune response via activating ER stress in the rat prefrontal cortex. Curr Med Sci 41(4):788–802 34403105 10.1007/s11596-021-2401-7

[42] Bonaventura P, Lamboux A, Albarède F, Miossec P (2017) Regulatory effects of zinc on cadmium-induced cytotoxicity in chronic inflammation. PLoS ONE 12(7):e0180879 28742830 10.1371/journal.pone.0180879 PMC5526586

[43] Ni HJ, Liu FF, Liang X, Yin YL, Liu G (2020) The role of zinc chelate of hydroxy analogue of methionine in cadmium toxicity: effects on cadmium absorption on intestinal health in piglets. Animal 14(7):1382–1391 32051055 10.1017/S1751731120000166

[44] Contreras-Zentella ML, Hernández-Muñoz R (2016) Is liver enzyme release really associated with cell necrosis induced by oxidant stress? Oxid Med Cell Longev 1:1–12 10.1155/2016/3529149 PMC4699024 26798419

[45] Elbakary RH (2017) Histological study of the effects of olanzapine on the liver of adult male albino rat with and without vitamin C. Egypt J Histol 40(1):1–11

[46] Mustari A, Alam M, Miah MA, Sujan KM, Mahamud AGMSU, Chowdhury EH (2023) Restoration of hepatorenal dysfunction and injury by zinc and folic acid combination in bisphenol A-intoxicated mice. J Adv Biotechnol Exp Ther 6(3):541–551

[47] Bolkent S, Arda-Pirincci P, Bolkent S, Yanardag R, Tunali S, Yildirim S (2006) Influence of zinc sulfate intake on acute ethanol-induced liver injury in rats. World J Gastroenterol 12(27):4345–4351 16865776 10.3748/wjg.v12.i27.4345 PMC4087745

[48] Aboulthana WM, Ibrahim NE, Hussien AG, Hassan AK, Khalil WKB, Abdel-Gawad H, Taha HA, Kelany AK, Ahmed KA (2025) Assessment of the gold nanoparticles biosynthesized using *Casuarina equisetifolia* bark extract against the ethion induced hepato- and neurotoxicity in rats. J Genet Eng Biotechnol 23(2):100495 40390491 10.1016/j.jgeb.2025.100495 PMC12060469

[49] El-Feky AM, Aboulthana WM, El-Rashedy AA (2024) Assessment of the in vitro anti-diabetic activity with molecular dynamic simulations of limonoids isolated from Adalia lemon peels. Sci Rep 14(21478):1–19 39277638 10.1038/s41598-024-71198-5 PMC11401861

[50] Aboulthana WM, Ibrahim NE, Hassan AK, Bassaly WK, Abdel-Gawad H, Taha HA, Ahmed KA (2023) The hepato- and neuroprotective effect of gold *Casuarina equisetifolia* bark nano-extract against Chlorpyrifos-induced toxicity in rats. J Genet Eng Biotechnol 21:158 38040926 10.1186/s43141-023-00595-6 PMC10692062

[51] Abdou HM, Yousef MI, Newairy AA (2018) Triton WR-1339-induced hyperlipidemia, DNA fragmentation, neurotransmitters inhibition, oxidative damage, histopathological and morphometric changes: the protective role of soybean oil. JoBAZ 79:51

[52] Adams BM, Canniff NP, Guay KP, Hebert DN (2021) The role of endoplasmic reticulum chaperones in protein folding and quality control. Prog Mol Subcell Biol 59:27–50 34050861 10.1007/978-3-030-67696-4_3 PMC9185992

[53] El-Sayed AB, Aboulthana WM, El-Feky AM, Ibrahim NE, Seif MM (2018) Bio and phyto-chemical effect of *Amphora coffeaeformis* extract against hepatic injury induced by paracetamol in rats. Mol Biol Rep 45(6):2007–2023 30244397 10.1007/s11033-018-4356-8

[54] El-Shamarka MEA, Aboulthana WM, Omar NI, Mahfouz MM (2024) Evaluation of the biological efficiency of *Terminalia chebula* fruit extract against neurochemical changes induced in brain of diabetic rats: an epigenetic study. Inflammopharmacology 32:1439–1460 38329710 10.1007/s10787-024-01428-9 PMC11006788

[55] Aboulthana WM, Ibrahim NE, Osman NM, Seif MM, Hassan AK, Youssef AM, El-Feky AM, Madboli AA (2020) Evaluation of the biological efficiency of silver nanoparticles biosynthesized using Croton tiglium L. seeds extract against azoxymethane induced colon cancer in rats. Asian Pac J Cancer Prev 21(5):1369–1389 32458646 10.31557/APJCP.2020.21.5.1369 PMC7541879

[56] Aboulthana WM, Shousha WG, Essawy EA, Saleh MH, Salama AH (2021) Assessment of the anti-cancer efficiency of silver *Moringa oleifera* leaves nano-extract against colon cancer induced chemically in rats. Asian Pac J Cancer Prev 22(10):3267–3286 34711004 10.31557/APJCP.2021.22.10.3267 PMC8858244

[57] Gatiatulina ER, Sheina EA, Nemereshina ON, Popova EV, Polyakova VS, Agletdinov EF, Sinitskii AI, Skalny AV, Nikonorov AA, Tinkov AA (2020) Effect of Zn supplementation on trace element status in rats with diet-induced non-alcoholic fatty liver disease. Biol Trace Elem Res 197:202–212 31832925 10.1007/s12011-019-01985-z

[58] Brzóska MM, Kozłowska M, Rogalska J, Gałażyn-Sidorczuk M, Roszczenko A, Smereczański NM (2021) Enhanced zinc intake protects against oxidative stress and its consequences in the brain: a study in an *in vivo* rat model of cadmium exposure. Nutrients 13(2):478 33572579 10.3390/nu13020478 PMC7911633

[59] Narayanan SE, Rehuman NA, Harilal S, Vincent A, Rajamma RG, Behl T, Uddin MS, Ashraf GM, Mathew B (2020) Molecular mechanism of zinc neurotoxicity in Alzheimer’s disease. Environ Sci Pollut Res 27:43542–43552 10.1007/s11356-020-10477-w 32909132

[60] De Freitas RB, Augusti PR, De Andrade ER, Rother FC, Rovani BT, Quatrin A, Alves NM, Emanuelli T, Bauermann LFJ (2014) Black grape juice protects spleen from lipid oxidation induced by gamma radiation in rats. Food Biochem 38:119–127

[61] Aboulthana WM, Madboli AA, Hussien AG, Seif M (2024) Exploring the protective effect of silver *Croton tiglium* nano-extract against azoxymethane induced toxicity in female reproductive organs in rats. Heliyon 10(21):e38820 39524721 10.1016/j.heliyon.2024.e38820 PMC11550675

[62] Al-Enazi MM (2014) Combined therapy of rutin and silymarin has more protective effects on streptozotocin-induced oxidative stress in rats. J Appl Pharm Sci 4(01):021–028

[63] Seif MM, Ahmed-Farid OA, Aboulthana WM (2017) Evaluation of the protective effect of *Acacia senegal* extract against di-(2-ethylhexyl phthalate) induced hepato- and neurotoxicity in rats. Annu Res Rev Biol 19(2):1–17

[64] Benjamin S, Pradeep S, Josh MKS, Kumar S, Masai E (2015) A monograph on the remediation of hazardous phthalates. J Hazard Mater 298:58–72 26004054 10.1016/j.jhazmat.2015.05.004

[65] Udia PM, Takem LP, Ufot UF, Antai AB, Owu DU (2016) Insulin and alpha amylase levels in alloxan-induced diabetic rats and the effect of Rothmannia hispida (K. Schum) Fagerl leaf extract. J Phytopharmacol 5:1–5

[66] Aboulthana WM, El-Feky AM, Ibrahim NE, Sahu RK, El-Sayed AB (2018) Evaluation of the pancreatoprotective effect of *Nannochloropsis oculata* extract against streptozotocin-induced diabetes in rats. J Appl Pharmac Sci 8(06):046–058

[67] Tupe RS, Tupe SG, Tarwadi KV, Agte VV (2010) Effect of different dietary zinc levels on hepatic antioxidant and micronutrients indices under oxidative stress conditions. Metabolism 59(11):1603–1611 20359724 10.1016/j.metabol.2010.02.020

[68] Siddique W, Rashid N, Asim S, Firdous A, Siddique H, Hanif A, Fatima A (2021) Comparison of histoprotective effect of silymarin and zinc sulfate against hepatotoxicity induced by isoniazid and rifampicin combination in animal model. Pak J Med Health Sci 15(1):73–78

[69] Nazarizadeh A, Asri-Rezaie S (2016) Comparative study of antidiabetic activity and oxidative stress induced by zinc oxide nanoparticles and zinc sulfate in diabetic rats. AAPS PharmSciTech 17:834–843 26349687 10.1208/s12249-015-0405-y

[70] Huang XF, Song X (2019) Effects of antipsychotic drugs on neurites relevant to schizophrenia treatment. Med Res Rev 39:386–403 29785841 10.1002/med.21512

[71] Kowalchuk C, Kanagasundaram P, McIntyre WB, Belsham DD, Hahn MK (2019) Direct efects of antipsychotic drugs on insulin, energy sensing and infammatory pathways in hypothalamic mouse neurons. Psychoneuroendocrinology 109:104400 31404896 10.1016/j.psyneuen.2019.104400

[72] Turkheimer FE, Selvaggi P, Mehta MA, Veronese M, Zelaya F, Dazzan P, Vernon AC (2020) Normalizing the abnormal: do antipsychotic drugs push the cortex into an unsustainable metabolic envelope? Schizophr Bull 46(3):484–495 31755955 10.1093/schbul/sbz119 PMC7147598

[73] Martínez MA, Rodríguez JL, Lopez-Torres B, Martínez M, Martínez-Larrañaga MR, Maximiliano JE, Anadón A, Ares I (2020) Use of human neuroblastoma SH-SY5Y cells to evaluate glyphosate-induced effects on oxidative stress, neuronal development and cell death signaling pathways. Environ Int 135:105414 31874349 10.1016/j.envint.2019.105414

[74] Li T, Bonkovsky HL, Guo J (2011) Structural analysis of heme proteins: implications for design and prediction. BMC Struct Biol 11:13 21371326 10.1186/1472-6807-11-13 PMC3059290

[75] Jahr JS, Guinn NR, Lowery DR, Shore-Lesserson L, Shander A (2021) Blood substitutes and oxygen therapeutics: a review. Anesth Analg 132:119–129 30925560 10.1213/ANE.0000000000003957

[76] Chen L, Yang Z, Liu H (2023) Hemoglobin-based oxygen carriers. Medicina 59(2):396 36837597 10.3390/medicina59020396 PMC9962799

[77] Olechnowicz J, Tinkov A, Skalny A, Suliburska J (2018) Zinc status is associated with inflammation, oxidative stress, lipid, and glucose metabolism. J Physiol Sci 68(1):19–31 28965330 10.1007/s12576-017-0571-7 PMC5754376

[78] Cerofolini L, Fragai M, Luchinat C (2019) Mechanism and inhibition of matrix metalloproteinases. Curr Med Chem 26(15):2609–2633 29589527 10.2174/0929867325666180326163523

