# Supplementary material for: Zinc Sulfate Alleviates Olanzapine Induced Alteration in Hepatic Protein Patterns and Antioxidant Defense System in Rats
Source: Biol Trace Elem Res. 2025 May 30;204(1):189–205. doi: 10.1007/s12011-025-04673-3 (PMC12847163; doi:10.1007/s12011-025-04673-3)
Supplement: Supplementary file 1 — (DOC 2.01 MB) [file 12011_2025_4673_MOESM1_ESM.doc]

**Supplementary Table 1:** Composition of normal chow diet administered to all treated rats during the experiement.

| **Diet** | **Quantity** |
| --- | --- |
| **Energy composition (%)** | |
| Carbohydrate | 63.9% |
| Protein | 20.3% |
| Fat | 15.8% |
| **Ingredients (g/kg)** | |
| Casein | 200 |
| L-Cystine | 3 |
| Corn starch | 397 |
| Maltodextrin | 132 |
| Sucrose | 100 |
| Cellulose | 50 |
| Soybean Oil | 70 |
| t-Butylhydroquinone | 0.014 |
| Mineral Mix | 35 |
| Vitamin Mix | 10 |
| Choline Bitartrate | 2.5 |


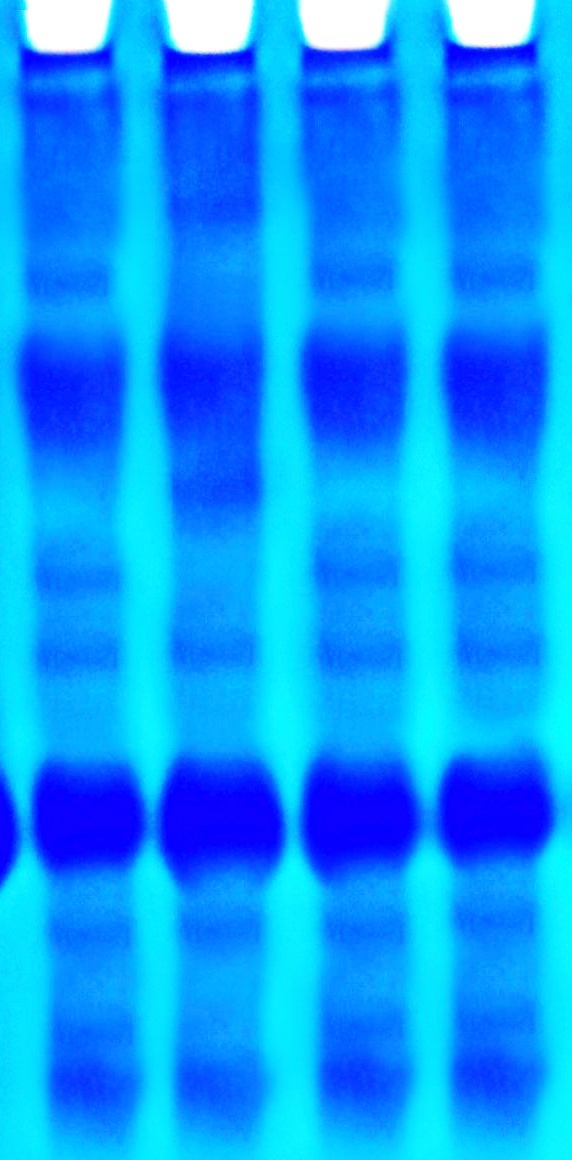


Unprocessed native protein pattern in liver tissue.


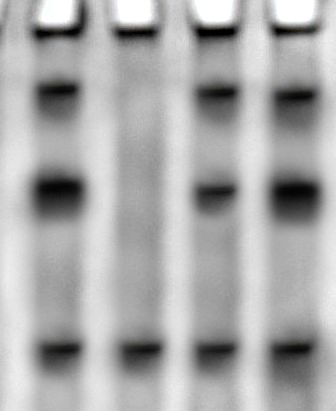


Unprocessed lipid moiety of native protein pattern in liver tissue.


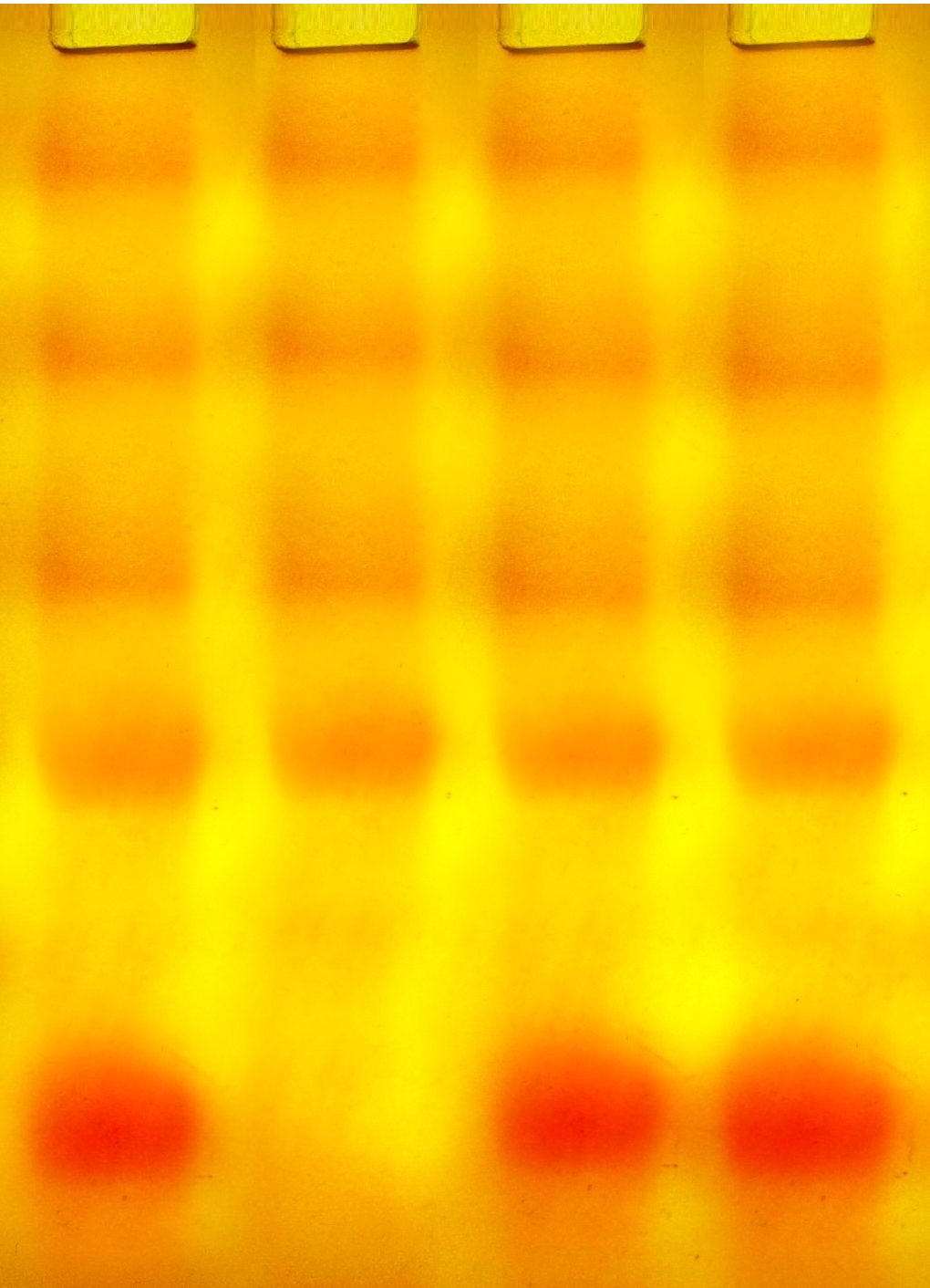


Unprocessed calcium moiety of native protein pattern in liver tissue.


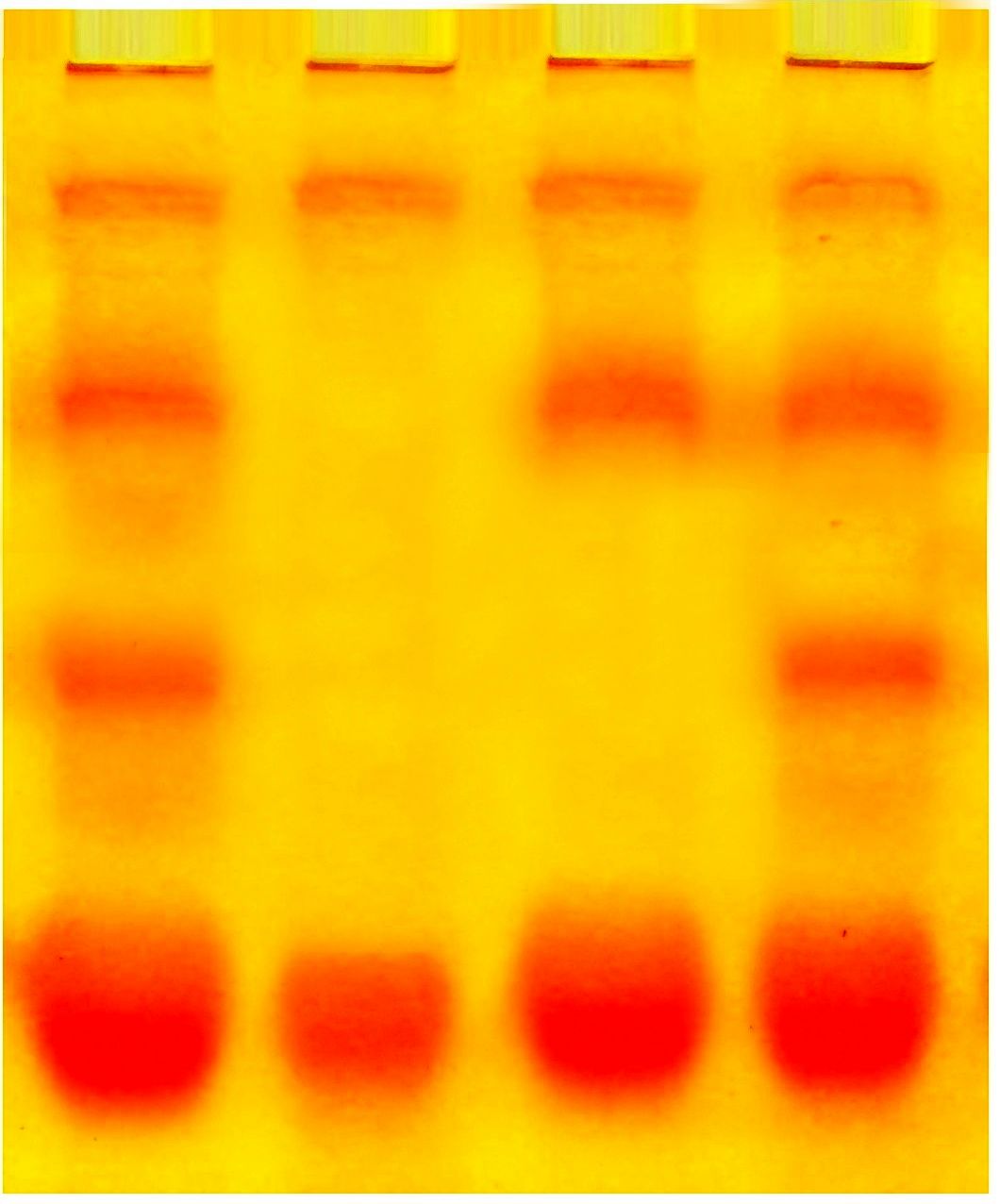


Unprocessed catalase (CAT) isoenzyme pattern in liver tissue.


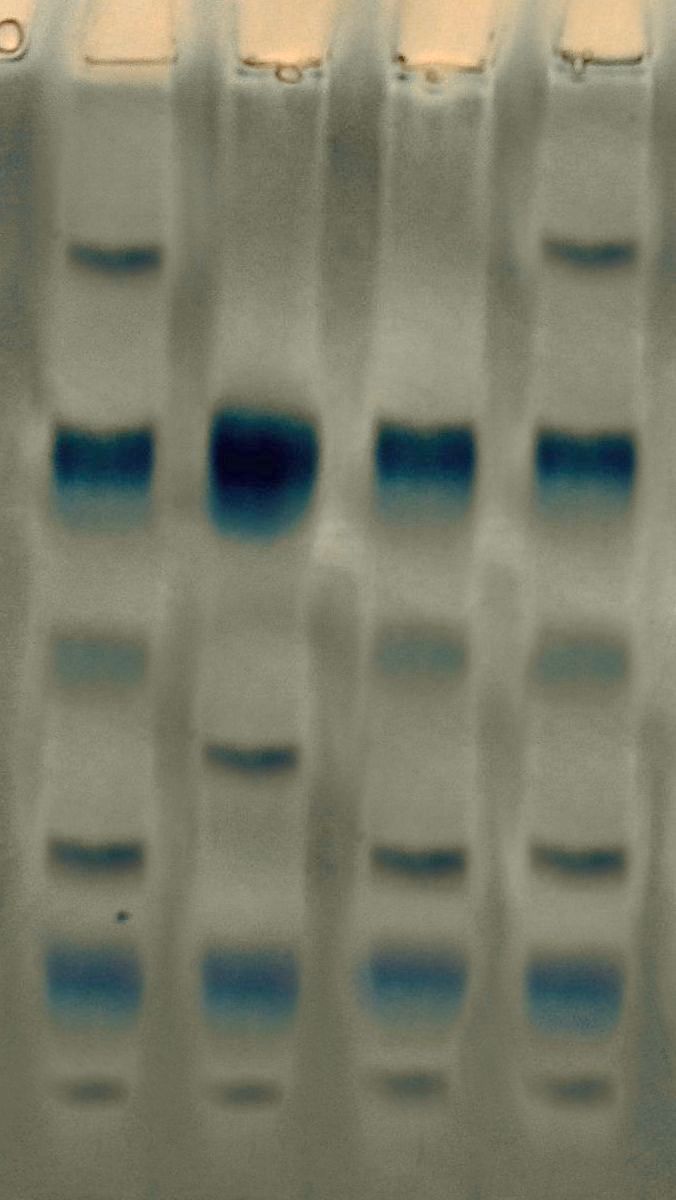


Unprocessed peroxidase (POX) isoenzyme pattern in liver tissue.


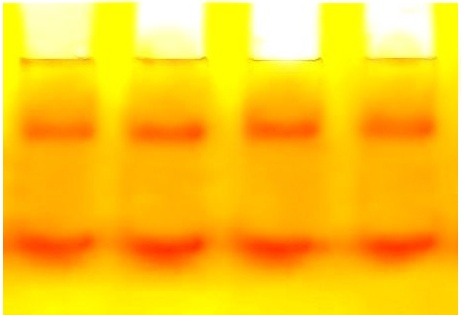


Unprocessed α-amylase isoenzyme pattern in liver tissue.


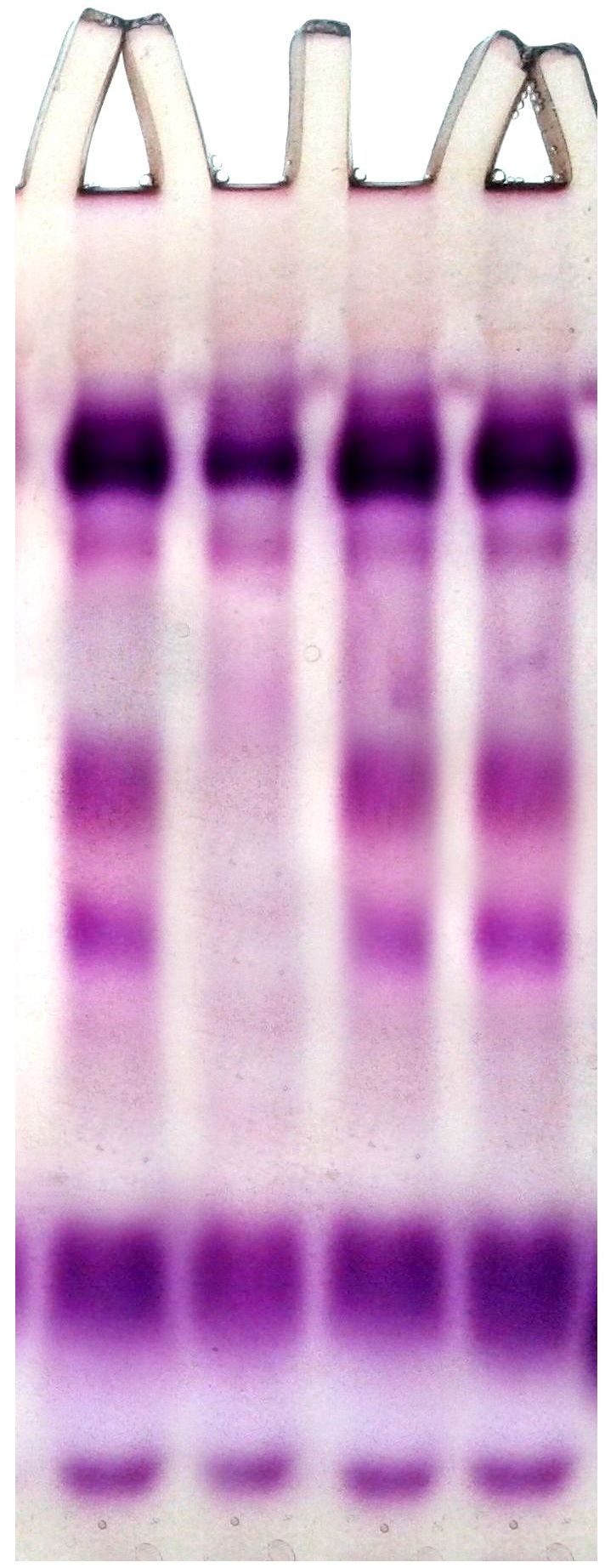


Unprocessed β-esterase (β-EST) isoenzyme pattern in liver tissue.
